# A New Switched State Jump Observer for Traffic Density Estimation in Expressways Based on Hybrid-Dynamic-Traffic-Network-Model

**DOI:** 10.3390/s19183822

**Published:** 2019-09-04

**Authors:** Wenbin Zha, Yuqi Guo, Huawei Wu, Miguel Angel Sotelo, Yulin Ma, Qian Yi, Zhixiong Li, Xin Sun

**Affiliations:** 1 Research Institute of Highway Ministry of Transport of China, Beijing 100088, China (W.Z.) (Y.G.) (Q.Y.) (X.S.); 2 Hubei Key Laboratory of Power System Design and Test for Electrical Vehicle & School of Automotive and Traffic Engineering, Hubei University of Arts and Science, Xiangyang 441053, China; 3 Department of Computer Engineering, University of Alcalá, Alcalá de Henares, 28801 Madrid, Spain; 4 Suzhou Automotive Research Institute, Tsinghua University, Suzhou 215134, China; 5 School of Mechanical, Materials, Mechatronic and Biomedical Engineering, University of Wollongong, Wollongong, NSW 2522, Australia

**Keywords:** traffic density estimation, traffic measurement, state jump observer, hybrid dynamic system

## Abstract

When faced with problems such as traffic state estimation, state prediction, and congestion identification for the expressway network, a novel switched observer design strategy with jump states is required to reconstruct the traffic scene more realistically. In this study, the expressway network is firstly modeled as the special discrete switched system, which is called the piecewise affine system model, a partition of state subspace is introduced, and the convex polytopes are utilized to describe the combination modes of cells. Secondly, based on the hybrid dynamic traffic network model, the corresponding switched observer (including state jumps) is designed. Furthermore, by applying multiple Lyapunov functions and S-procedure theory, the observer design problem can be converted into the existence issue of the solutions to the linear matrix inequality. As a result, a set of gain matrices can be obtained. The estimated states start to jump when the mode changes occur, and the updated value of the estimated state mainly depends on the estimated and the measured values at the previous time. Lastly, the designed state jump observer is applied to the Beijing Jingkai expressway, and the superiority and the feasibility are demonstrated in the application results.

## 1. Introduction

Throughout past decades, traffic flow modeling has always been a hot topic among scholars, and various models are gradually being applied to solve traffic flow analysis, control, state estimation, and prediction. In particular, dynamic model-based traffic state estimator design technology has proven its usefulness in solving traffic state estimation, reconstruction, and prediction. In recent years, with the continuous progress and development of technologies, a number of new state estimators have been designed and are gradually being applied to solve the state estimation problem of traffic networks with different sizes and topology structures. State observers and Kalman filters based on the cell transmission model (CTM) [1,2] and various improved cell transmission models are the most popular in current studies.

Sun et al. used the Kalman filters to estimate traffic state [3,4]. Canudas-de-Wit et al. [5] improved the CTM by using the called graph constrained method, and, based on this new model, a state observer was applied to a real freeway. Further, a robust approach was considered to improve the estimation accuracy in reference [6]. Chen et al. proposed a dynamic graph hybrid automata modeling framework [7,8] by combining the dynamic graph [9] with the hybrid automata theory [10,11] and deduced the piecewise affine system model (PWASM) of the traffic network [12]. Based on the PWASM, a series of switched state observers [13,14,15,16,17] and Kalman filters [18] were studied to solve the issues of traffic density estimation and congestion identification for traffic networks of any size and with any topology structures.

In addition, for other system models, corresponding observer design methods were also studied [19,20,21]. In reference [22], based on a hybrid 2-D model of the switched stochastic systems, the observer was designed to solve the tracking control problem. The reduced-order state observers were studied in literature [23,24,25]. In references [26,27,28], distributed state observers were applied to the large scale systems. The types of robust observers were proposed in the switched systems with unknown inputs [29] and discrete nonlinear systems with disturbances [30]. Other methods were also used in references [31,32].

Those studies mentioned above have shown that all the methods are only partially successful in accurately solving the problem of state estimation. All these approaches mainly rely on the strictly synchronous information from the actual system and the state observer system. Especially for the switched system, it is always assumed that there is no time delay in the switching signal. However, this assumption disobeys fact. As a typical switched system, there is always a time interval between the real traffic network system and its estimation system, either leading or lagging. That is to say, synchronous switching is almost impossible because of the inherent nature of the system.

As can be seen from the above analysis, it is not feasible to solve the state estimation problem of a real traffic network using the traditional observer design strategy. Therefore, discovering a more efficient method to solve this problem has become an important goal to pursue. For a similar issue, a state jump observer was presented for the continuous system in [33]. In a discrete system such as a traffic network system, the same observer design idea can be introduced.

In literature [20,21], the common Lyapunov function was used to solve the convergence of the error estimation systems. In this work, unlike most of the existing results, the state jump observer design is transformed to the linear matrix inequality problem by introducing Lyapunov function [34] and S-Prodecure [35], and the multiple Lyapunov functions are utilized to guarantee that the solution of the error system exists. The design of the observer can be completed to accurately reconstruct the real traffic states.

The organization of this paper is as follows. In Section 2, the hybrid dynamic model is deduced by embedding the CTM into the Dynamic Graph Hybrid Automata (DGHA) framework. In Section 3, the state jump observer is designed. In Section 4, the results obtained by applying the methods to the real expressway are illustrated. The paper is concluded in Section 5.

## 2. Hybrid Dynamic Model for Traffic Network

In previous work, a new macroscopic traffic flow modeling method on the basis of the well-known CTM was proposed to describe the evolution law of traffic flow over the road network. This method was named the Dynamic Graph Hybrid Automata (DGHA). In the model, the nonlinear description was transformed into the piecewise linear expression of the multi-mode switching among cells through the space partition principle, and the combined modes between cells could be described by the convex polytopes. The traffic network dynamical system can be described by a piecewise affine system with the following equation (for the detailed modeling process, the reader can refer to references [12,17]).

(1) $$\{\begin{array}{l} x(t+1)=A_{\sigma (t)}x(t)+Bu(t)+F_{\sigma (t)}\\ y(t)=Cx(t) \end{array}$$

where $x={\left[\rho_{1},\cdots ,\rho_{N}\right]}^{T}\in {\mathbb{R}}^{N}$ denotes the traffic density vector of the road network, the input vector $u\in {\mathbb{R}}^{M}$ is added to represent the traffic demand of the road network, $y(t)\in {\mathbb{R}}^{P}$ is the measured output vector of the sensors, $F_{\sigma (t)}$ is a vector consisting of the parameters in the fundamental diagrams of all the road segments, $A_{\sigma }$, B, and C are the system matrix, the input matrix, and the output matrix, respectively, σ(t) is the switching function, and $\sigma :[0,+\infty )\to I_{S}=\{1,2,\cdots ,S\}$ is determined by the convex polytopes $D_{s}$, i.e., σ(t)=s if and only if $x(t)\in D_{s}$.

**Figure 1:**
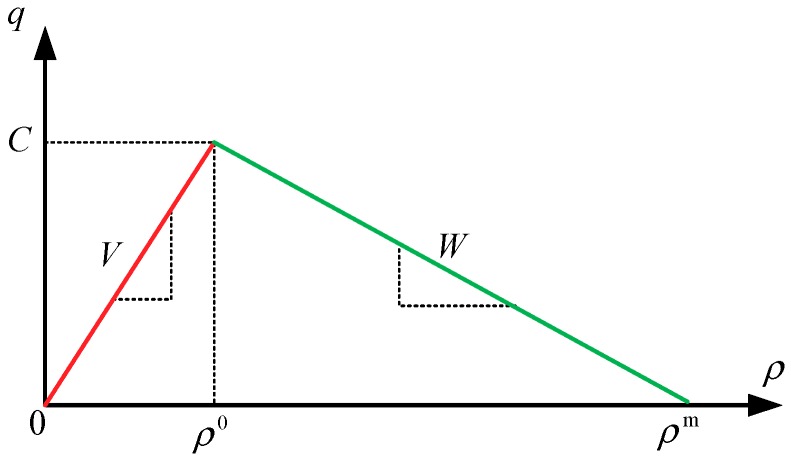
Triangular fundamental diagram.

In the model, the triangular fundamental diagram is used to approximately describe the relationship between the traffic flow and the density of the road segment (cell) (see Figure 1), where ρ is the traffic density (vehicles per kilometer), q is the traffic flow (vehicles per hour), C is the traffic capacity (vehicles per hour), V is the free flow speed (kilometers per hour), W is the traffic wave speed (kilometers per hour), $\rho^{0}$ is the critical density (vehicles per kilometer), and $\rho^{m}$ is the maximum/jam density (vehicles per kilometer).

## 3. Design of the State Jump Observer

In order to express the switching processes between different traffic flow modes as clearly as possible, the linear hyper planes following Equation (2) describing the changes between different modes (subsystems) are defined, and $D_{i,j}$ is called a switched set.

(2) $$D_{i,j}=\left\{x\left(t\right)\in {\mathbb{R}}^{N}|x\left(t\right)\in D_{i},x\left(t+1\right)\in D_{j},i,j\in S\right\}$$

where $D_{i}$ and $D_{j}$ denote the active field of the *i*th mode and the *j*th mode, respectively, which are described by the convex polytopes. The mode *i* cannot be switched to the mode *j*, if $D_{i,j}$ is an empty set.

**Remark** **1.** *Traffic flow network can be modeled to be the piecewise affine system by introducing the state subspace, each subsystem can be seen as a mode and can be described by the convex polytope, and the changes of the values between the adjacent modes can be expressed using linear hyper planes.*

**Figure 2:**
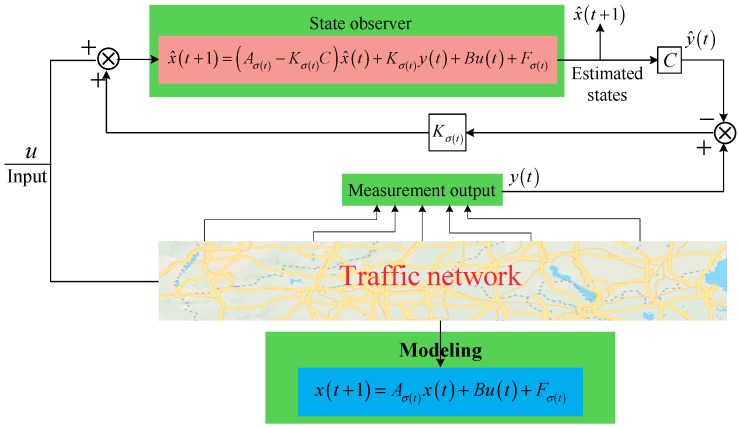
Structure of state synchronous observer.

**Figure 3:**
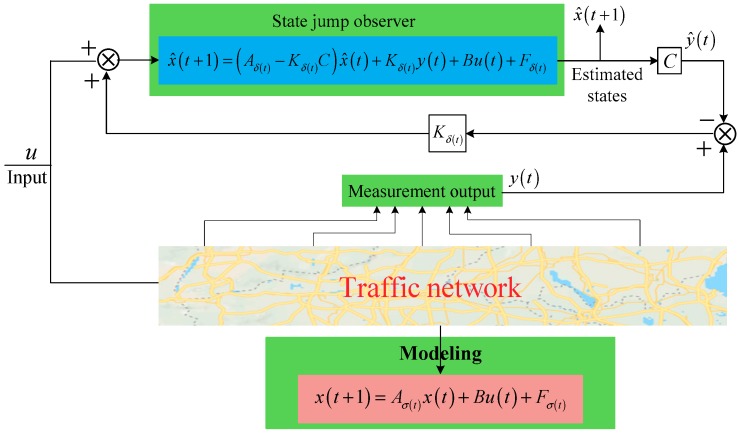
Structure of state jump observer.

For the convenience of design and computing, an important assumption is essential in the traditional switched observer design—the switching of the observer and the switching of the system are synchronous. However, strict synchronization is impossible for the real traffic network, thus this assumption does not hold. As a result, a novel structure observer, which is called state jump observer, is proposed. The structure diagrams of the switched state synchronous observer and the switched state jump observer are shown in Figure 2 and Figure 3, respectively.

**Definition** **1.** ***Switched State Synchronous Observer:** For the switched system, a state observer is called a synchronous one as the switching sequence changes if and only if the state switching of the observer system is synchronous with that of the original system, thus there is no time-delay.*

**Definition** **2.** ***Switched State Jump Observer:** For the switched system, a state observer is called a state jump one if and only if the state switching of the observer system is not synchronous with that of the original system, thus there is always time-delay as the switching sequence changes.*

Therefore, based on system (1), the switched state observer of the road network system can be described as the following Equation (3):

(3) $$\hat{x}(t+1)=\left(A_{\delta (t)}-K_{\delta (t)}C\right)\hat{x}(t)+K_{\delta (t)}y(t)+Bu(t)+F_{\delta (t)}$$

where $\hat{x}\left(t\right)\in {\mathbb{R}}^{N}$ is the estimated state vector, $K_{\delta }\in K≜\left\{K_{1},K_{2},K_{3},\cdots ,K_{S}\right\}$ is the observer gain matrices to be designed, and the switching function δ:[0,+∞)→{1,2,⋯,S} decides which one of the observer modes is active at a certain time point.

In order to describe the switching of the observer system, the same method as shown in Equation (2) can be used to describe the value changes of the switching function δ(t), which is as follows:

(4) $$D_{i,j}=\left\{\hat{x}\left(t\right)\in {\mathbb{R}}^{N}|\hat{x}\left(t\right)\in D_{i},\hat{x}\left(t+1\right)\in D_{j},i,j\in S\right\}$$

For the general linear time invariant system, which only includes single mode, supposing that the system is observable or detectable, only the observer gain matrix *K* has to be designed to ensure that the matrix *A-KC* is Schur stable and that the estimated state $\hat{x}$ is able to track the system state x. Similarly, for the traffic network system, which is a multi-mode switching, it seems that a series of observer gain matrices need to be computed such that each of the *A_i_-K_i_C* is Schur stable. If the active mode of the system (1) is known, it is only to activate the corresponding observer mode. However, there is no guarantee the estimation error will converge by using this method, even if the estimation error of each mode is convergent—not to mention the active mode of the traffic network is unknown. Thus, the classical observer design approach is no longer applicable, and a novel methodology for the piecewise affine switched linear system (1) has to be investigated.

In the existing literature (including our previous work), most state observers for switched systems are designed in an ideal case. In references [12,13,14,15,16,17], it is assumed that the switching of the observer system is synchronous with that of the original system modes in the section of observer design. However, it is well known that this assumption is an ideal case; it does not conform to the actual situation. In most practical cases, compared to the original system (1), the switching between modes of the observer system (3) is always ahead or delayed. It is difficult to guarantee the estimation errors converge for the ideal assumption. Based on the analysis above, the switched observer including state jumps is adopted to solve the issue of traffic density estimation.

The state estimation error dynamics are obtained by combining the system (1) and the observer system (3).

(5) $$e\left(t+1\right)=\bar{A}e\left(t\right)+\Delta Ax\left(t\right)+\Delta F$$

where $\bar{A}=A_{\delta \left(t\right)}-L_{\delta \left(t\right)}C$, $\Delta A=A_{\sigma \left(t\right)}-A_{\delta \left(t\right)}$, $\Delta F=F_{\sigma \left(t\right)}-F_{\delta \left(t\right)}$.

In this paper, the multiple Lyapunov functions method is introduced to solve the stability of the error dynamic system, and each Lyapunov function can be expressed as $V_{i}\left(e\right)=e^{T}P_{i}e$. Thus, the change of the energy can be obtained as follows:

(6) $$\begin{array}{cc} \Delta V_{i}\left(e\right) & =V_{\left[e_{i}\left(t+1\right)\right]}-V_{\left[e_{i}\left(t\right)\right]}\\ & ={\left[{\bar{A}}_{i}e+\Delta A_{i}x+\Delta F_{i}\right]}^{T}P\left[{\bar{A}}_{i}e+\Delta A_{i}x+\Delta F\right]-e^{T}P_{i}e\\ & =e^{T}\left({\bar{A}}_{i}^{T}P_{i}{\bar{A}}_{i}-P_{i}\right)e+e^{T}{\bar{A}}_{i}^{T}P_{i}\Delta A_{i}x+e^{T}{\bar{A}}_{i}^{T}P_{i}\Delta F_{i}+x^{T}\Delta A_{i}^{T}P_{i}{\bar{A}}_{i}e\\ & \;+x^{T}\Delta A_{i}^{T}P_{i}\Delta A_{i}x+x^{T}\Delta A_{i}^{T}P_{i}\Delta F_{i}+\Delta F_{i}^{T}P_{i}{\bar{A}}_{i}e+\Delta F_{i}^{T}P_{i}\Delta A_{i}x+\Delta F_{i}^{T}P_{i}\Delta F_{i} \end{array}$$

where ${\bar{A}}_{i}=A_{i}-L_{i}C$, $\Delta A_{i,j}=A_{j}-A_{i}$, $\Delta F_{i,j}=F_{j}-F_{i}$. Each $P_{i}\in {\mathbb{R}}^{N}$ is the symmetric positive definite matrix. The state of the observer system and the state of the origin system evolve according to mode *i* and mode *j*, respectively.

Next, a theorem is given for reference:

**Theorem** **1** [33,34]**.** *The state estimation error dynamics (5) is eventually bounded by* $e_{\max}$, *and* x *is eventually bounded by* $x_{\max}$*, if there exist the matrices* $P_{i},L_{i}$ *and the nonnegative constant* μ, $\lambda_{i,j}$*, such that the matrix inequality (7) is satisfied.*

(7) $$\Phi_{i,j}=\left[\begin{array}{ccc} \Phi_{i,j}^{11} & \Phi_{i,j}^{12} & \Phi_{i,j}^{13}\\ {\left(\Phi_{i,j}^{12}\right)}^{T} & \Phi_{i,j}^{22} & \Phi_{i,j}^{23}\\ {\left(\Phi_{i,j}^{13}\right)}^{T} & {\left(\Phi_{i,j}^{23}\right)}^{T} & \Phi_{i,j}^{33} \end{array}\right]\leq 0,\;i,j\in S$$

where $\Phi_{i,j}^{11}={\bar{A}}_{i}^{T}P_{i}{\bar{A}}_{i}-P_{i}-I$, $\Phi_{i,j}^{12}={\bar{A}}_{i}^{T}P_{i}\Delta A_{i}$, $\Phi_{i,j}^{13}={\bar{A}}_{i}^{T}P_{i}\Delta F_{i}$, $\Phi_{i,j}^{22}=\Delta A_{i}^{T}P\Delta A_{i}+\lambda_{i,j}\mu_{i}^{2}I$, $\Phi_{i,j}^{23}=\Delta A_{i}^{T}P\Delta F_{i}$, $\Phi_{i,j}^{33}=\Delta F_{i}^{T}P_{i}\Delta F_{i}$.

The detailed proof process is as follows.

**Proof** **of** **Theorem** **1.** The constant piecewise Lyapunov function $V_{i}\left(e\right)$ is expressed as:

(8) $$V_{i}\left(e\right)=e^{T}P_{i}e$$

where $P_{i}$ is the symmetric positive definite matrix, and:

(9) $$\begin{array}{cc} \Delta V_{i}\left(e\right) & =V_{\left[e_{i}\left(t+1\right)\right]}-V_{\left[e_{i}\left(t\right)\right]}\\ & ={\left[{\bar{A}}_{i}e+\Delta A_{i}x+\Delta F_{i}\right]}^{T}P\left[{\bar{A}}_{i}e+\Delta A_{i}x+\Delta F\right]-e^{T}P_{i}e\\ & =e^{T}\left({\bar{A}}_{i}^{T}P_{i}{\bar{A}}_{i}-P_{i}\right)e+e^{T}{\bar{A}}_{i}^{T}P_{i}\Delta A_{i}x+e^{T}{\bar{A}}_{i}^{T}P_{i}\Delta F\\ & \;+x^{T}\Delta A_{i}^{T}P_{i}{\bar{A}}_{i}e+x^{T}\Delta A_{i}^{T}P_{i}\Delta A_{i}x+x^{T}\Delta A_{i}^{T}P_{i}\Delta F\\ & \;+\Delta F_{i}^{T}P_{i}{\bar{A}}_{i}e+\Delta F_{i}^{T}P_{i}\Delta A_{i}x+\Delta F_{i}^{T}P_{i}\Delta F_{i} \end{array}$$

In order to ensure the error estimation system (5) is converged, the following inequality with a constraint condition has to be satisfied:

(10) $$\{\begin{array}{l} \Delta V_{i}\left(e\right)=V_{\left[e_{i}\left(t+1\right)\right]}-V_{\left[e_{i}\left(t\right)\right]}\\ \left\|e_{i}\right\|⩽\mu_{i}\left\|x_{i-\max}\right\| \end{array}$$

By using the S-Procedure [36], there must exist a nonnegative constant $\lambda_{i,j}$ such that the following inequality (11) is satisfied. Equation (9) with the constraint condition is equivalent to the following inequality for a nonnegative constant $\lambda_{i,j}$:

(11) $$\left\{\Psi^{T}P\Psi -e^{T}P_{i}e\right\}-\lambda_{i,j}\left(e^{T}e-\mu_{i}^{2}x^{T}x\right)⩽0$$

where $\Psi ={\bar{A}}_{i}e+\Delta A_{i,j}x+\Delta F_{i,j}$.

Relying on the Schur complement, the inequality (11) can be rewritten as follows:

(12) $${\left[\begin{array}{c} e\\ x\\ 1 \end{array}\right]}^{T}\left[\begin{array}{ccc} \Phi_{i,j}^{11} & \Phi_{i,j}^{12} & \Phi_{i,j}^{13}\\ {\left(\Phi_{i,j}^{12}\right)}^{T} & \Phi_{i,j}^{22} & \Phi_{i,j}^{23}\\ {\left(\Phi_{i,j}^{13}\right)}^{T} & {\left(\Phi_{i,j}^{23}\right)}^{T} & \Phi_{i,j}^{33} \end{array}\right]\left[\begin{array}{c} e\\ x\\ 1 \end{array}\right]⩽0$$

namely:

(13) $$\Phi_{i,j}=\left[\begin{array}{ccc} \Phi_{i,j}^{11} & \Phi_{i,j}^{12} & \Phi_{i,j}^{13}\\ {\left(\Phi_{i,j}^{12}\right)}^{T} & \Phi_{i,j}^{22} & \Phi_{i,j}^{23}\\ {\left(\Phi_{i,j}^{13}\right)}^{T} & {\left(\Phi_{i,j}^{23}\right)}^{T} & \Phi_{i,j}^{33} \end{array}\right]\leq 0,\;i,j\in S$$

It is noted that the inequality (13) is not a Linear Matrix Inequality (LMI) because it contains bilinearities, but it can be transformed to an LMI by employing the change of the variable where the transformed problem is now of LMI variety, thus the matrix inequality is obtained (7).

The proof is completed. □

For the observer design of the switched system, how to properly update the estimated states of the observer system when the observer mode changes at the switching sets (4) is another important problem, because it affects the observation accuracy directly.

The new estimated state of the observer can be obtained after jumping by the following equation:

(14) $${\hat{x}}^{\prime }(t+1)=\eta_{1}\hat{x}(t)+\eta_{2}y(t),\;\hat{x}(t)\in D_{i,j}$$

where ${\hat{x}}^{\prime }(t+1)$ is the updated state of the observer state $\hat{x}(t)$, and $\eta_{1}$ and $\eta_{2}$ are two coefficients that need to be computed to guarantee that the estimated error e is bound.

On the basis of Equation (14), the computational formula of the updated value of the observer is given as shown in Lemma 1.

**Lemma** **1.** *The updated state*${\hat{x}}^{\prime }$*can be obtained by the following equation:*

(15) $${\hat{x}}^{\prime }=\left[I-Q_{i}^{-1}{\left(CQ_{i}^{-1}\right)}^{†}C\right]\hat{x}+Q_{i}^{-1}{\left(CQ_{i}^{-1}\right)}^{†}y$$

where $\eta_{1}=I-Q_{i}^{-1}{\left(CQ_{i}^{-1}\right)}^{†}C$, $\eta_{2}=Q_{i}^{-1}{\left(CQ_{i}^{-1}\right)}^{†}$, $x^{†}$ denotes the pseudo-inverse of x.

**Remark** **2.** *As mentioned above,*$P_{i}$*is the symmetric positive definite matrix. Thus, there exist*$\Lambda_{i}$, $K_{i}$, $Q_{i}$*such that*$P_{i}=K_{i}\Lambda_{i}K_{i}^{T}$*and*$P_{i}=Q_{i}Q_{i}^{T}$, *where*$K_{i}$*and*$\Lambda_{i}$*are the orthonormal eigenvectors, and the diagonal matrix consists of the eigenvalues of*$P_{i}$. $Q_{i}$*is the symmetric positive definite matrix, and*$Q_{i}$*is not the only one. Eventually,*$Q_{i}=K_{i}\sqrt{\Lambda_{i}}K_{i}^{T}$.

**Remark** **3.** *In practice, the estimated state*$\hat{x}$*will abruptly jump to the new state*${\hat{x}}^{\prime }$*, once the*$\hat{x}$*reaches the linear hyper planes*$D_{i,j}$. ${\hat{x}}^{\prime }$*is called the updated density of*$\hat{x}$.

The detailed algorithm steps are as follows.

**Step 1:** Compute the matrices $A_{i}$, B, and $F_{i}$ and design the output matrix C such that the pair $\left(A_{i},C\right)$ is observable or detectable. If the system is not observable or detectable, the matrix C is redesigned until the system meets the conditions.

**Step 2:** Compute matrices $P_{i}$, and then calculate $Q_{i}$ by factorizing $P_{i}$; meanwhile, collect measurements y.

**Step 3:** Update the state value of the observer at the beginning of each mode by ${\hat{x}}^{\prime }=\left[I-Q_{i}^{-1}{\left(CQ_{i}^{-1}\right)}^{†}C\right]\hat{x}+Q_{i}^{-1}{\left(CQ_{i}^{-1}\right)}^{†}y$.

## 4. Case Study: Beijing Jingkai Expressway

### 4.1. Experiment Parameters Setting

**Figure 4:**
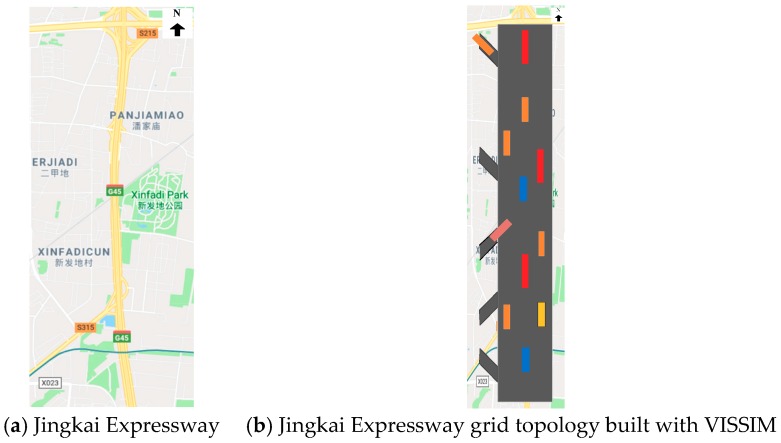
Experimental road section. (**a**) Jingkai Expressway; (**b**) Jingkai Expressway grid topology built with VISSIM.

In this section, an experiment example is presented to demonstrate the validity and the practicability of our approach by applying the designed state jump observer to the Beijing Jingkai expressway. The selected road section (see Figure 4a) is considered only from north to south, which is from the Majialou bridge to the Xihongmen toll station. The section is approximately 3.5 km long and is made up by three lanes. In accordance with the segment partition rules mentioned in reference [17], the road section is divided into six links including 14 cells, and the detailed results are labeled in Table 1.

First of all, according to the actual road network structure, the experiment road section is reconstructed by using the traffic simulator VISSIM (see Figure 4b). In order to reconstruct the traffic densities of all the cells, the traffic flow evolution process from 16:00 to 20:00 is then further simulated. The sample time interval is 5 s. Because there are three on-ramps and two off-ramps, the corresponding dimensions of the input matrix ***B*** are 14 × 3, and *b*_2,1_ = 0.0203, *b*_8,2_ = 0.0192, *b*_14,3_ = 0.0192, and others are 0.

According to the observability analysis of the traffic network, which is mentioned in references [17,36,37], and to ensure that the system is observable or detectable, the traffic detectors are placed in cells 1, 2, 3, 7, 8, 9, 10, 11, 12, 13, and 14. Thus, the matrix *C* has the following forms: *c*_1,1_ = *c*_2,2_ = *c*_3,3_ = *c*_4,7_ = *c*_5,8_ = *c*_6,9_ = *c*_7,10_ = *c*_8,11_ = *c*_9,12_ = *c*_10,13_ = *c*_11,14_ =1, and other entries are 0.

**Remark** **4.** *In practice, the output matrix **C** is not unique; it can be designed in many different forms according to the location and the types of the traffic sensors as long as the system (**A**,**C**) is observed.*

**Remark** **5.** *In order to reconstruct the full state by using the designed observer, we must construct the C matrix so that the pair (A_s, C) is observable or detectable, which is the sufficient and necessary condition of observer design, where C is the matrix related to the number and the places of traffic detectors in the road segments (cells). This is the first step for the design observer. Thus, the detectors are installed in cells of most of the highway segments to collect traffic data to guarantee the pair (A_s, C) is observable or detectable.*

### 4.2. Analysis Results

Jingkai expressway is not only the main road connecting the south area of Beijing with the city center but also one of the important passageways for the areas outside Beijing to enter the city. As the main passway out of Beijng, it carries considerable traffic every day, especially during the evening rush hour. Therefore, the period of time from 16:00 to 20:00 is chosen for our experiment.

In order to further verify the performance of the state jump observer designed in this paper, the conventional synchronous observer is used to reconstruct the cell densities of the experimental road section as the first step.

**Figure 5:**
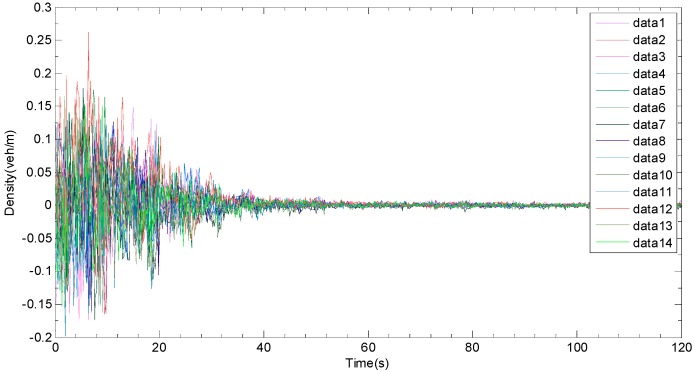
Error curve of synchronous observer.

**Figure 6:**
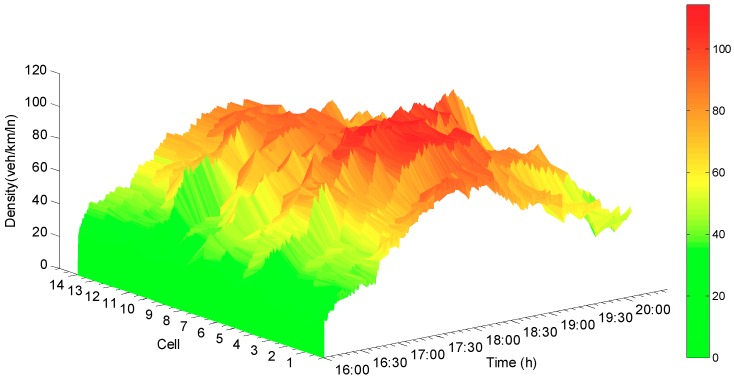
Real densities.

**Figure 7:**
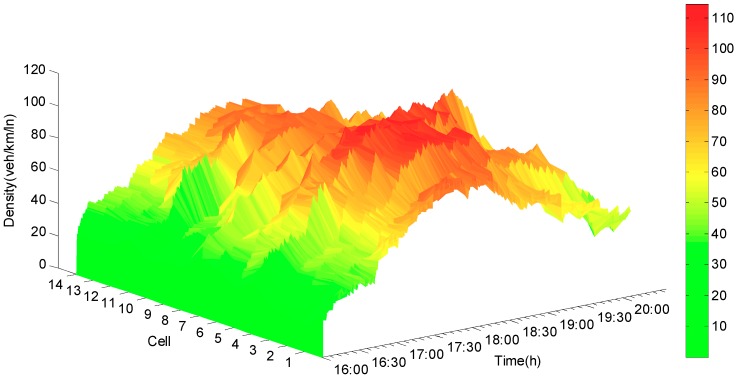
Estimated density of synchronous observer.

The Figure 5, Figure 6 and Figure 7 are the error curve, real densities and estimated densities, respectively From the error curve, which is shown in Figure 5, it can be seen that the estimation error between the real density and the estimated one begins to converge at about 30 s, and the convergence speed is acceptable. Since the observer and the actual road network system switch synchronously, the error does not jump and tends to be stable. Figure 7 indicates that the synchronous observer can accurately reconstruct the real density (see Figure 6).

**Figure 8:**
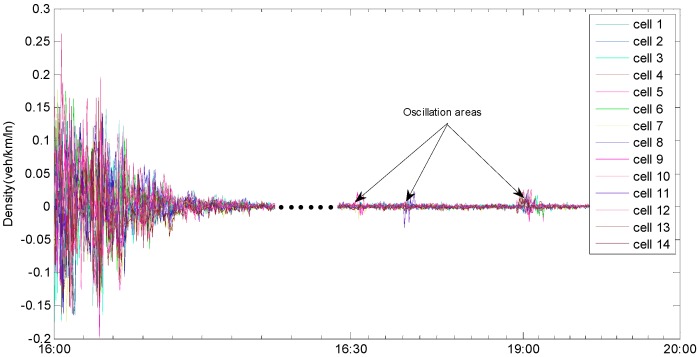
Error curve of state jump observer.

**Figure 9:**
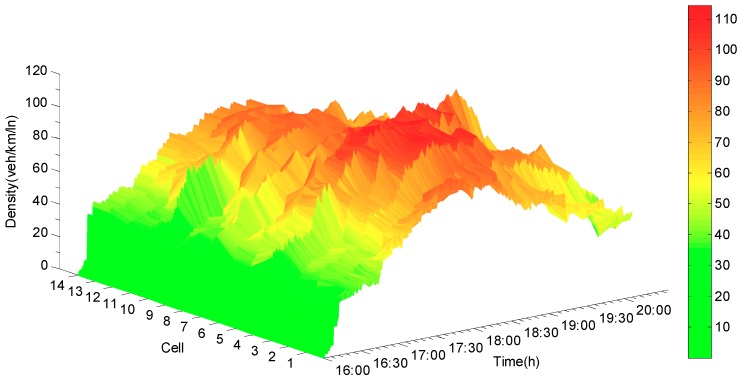
Estimated density of jump observer.

**Figure 10:**
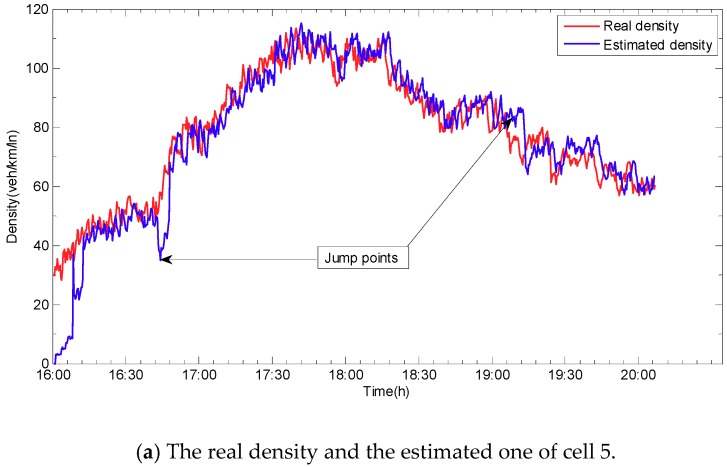

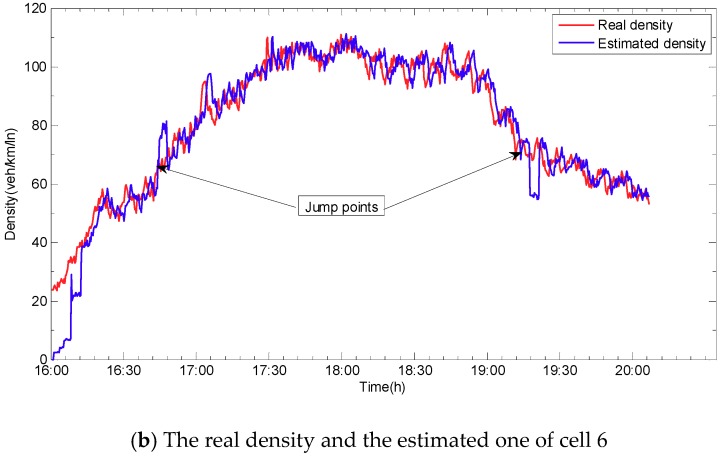
Experimental results of cell 5 and cell 6. (**a**) The real density and the estimated one of cell 5; (**b**) The real density and the estimated one of cell 6.

Compared to the synchronous state observer, the biggest difference is that the jumps and the oscillations appear when the mode changes occur in the error curve for the state jump observer (see Figure 8). The main reason is that, when the new mode is updated, the error system is oscillated repeatedly from the initial state until it converges again. The estimated densities, which are shown in Figure 9, exhibit that the densities can be reconstructed by using the state jump observer and that jumps only occur where the new models are updated. Figure 10 demonstrates the more detailed estimation results of cell 5 and cell 6. From the estimation results, the congested area and the congested period of the expressway can be easily identified. Thus, it can provide basis for residents to avoid traffic congestion and improve the road operation efficiency.

In addition, the Mean Percentage Error (MPE) and the Root Mean Squared Error (RMSE) are adopted to further verify the performance of the jump observer, which are shown in Equations (16) and (17), respectively.

(16) $$\mathrm{MPE}=\frac{1}{n}\sum_{i=1}^{n}\left|\frac{\rho_{i}\left(t\right)-{\hat{\rho }}_{i}\left(t\right)}{\rho_{i}\left(t\right)}\right|$$

(17) $$\mathrm{RMSE}=\sqrt{\frac{1}{n}\sum_{i=1}^{n}{\left[\rho_{i}\left(t\right)-{\hat{\rho }}_{i}\left(t\right)\right]}^{2}}$$

where $\rho_{i}\left(t\right)$ and ${\hat{\rho }}_{i}\left(t\right)$ are, respectively, the real densities and the estimated ones, and n is the total number of observations.

By using Equations (16) and (17), the MPE and the RMSE are computed for the two types of state observer, which are the synchronous state observer [16] and the state jump observer. The detailed results are shown in Table 2, Table 3, Table 4 and Table 5, respectively. From the results, we can see that, not only can the real densities be more accurately reconstructed by using the state jump observer, but the MPE and the RMSE of the state jump observer are better than the synchronous state observer as well.

**Remark** **6.** *For the synchronous state observer, the signal switching is synchronous between the observer system and the actual traffic network system without any time delay and advance. Therefore, it is unnecessary to consider the influence of the modes switching on the estimation accuracy when the system state is reconstructed by using this type of observer. On the contrary, the state of the jump observer cannot keep synchronization with the network system when cell mode changes occur, thus there may exist a time advance or a time delay. Therefore, when a new mode appears, the estimated state must jump and oscillate until the error between the observer and the actual system converges again.*

**Remark** **7.** *In order to reduce the complexity of observer design, the mode changes are ignored in the previous method, and thus jumps and oscillations disappear. However, in our method, in order to reconstruct the real densities more accurately, we consider the mode changes. Compared to the previous method, the biggest difference is that the jumps and the oscillations appear when the mode changes occur in the error curve for the state jump observer. The main reason is that, when the new mode is updated, the error system is oscillated repeatedly from the initial state until it converges again—this is the essential attribute of the real traffic network. This attribute cannot be eliminated and can only be weakened by the corresponding approach optimization. Although the oscillations appear when the mode changes occur, the error system can stabilize quickly by using our method, and it does not affect the accuracy of the results; this is also the advantage of our method.*

## 5. Conclusions

In the paper, based on the hybrid dynamic traffic network model, a switched observer with state jumps was designed to achieve traffic density reconstruction and solve traffic density estimation and congestion identification. Considering the particularity of the switched system, multiple Lyapunov functions were applied—one for each mode. The S-Procedure method was also used in the design procedure of the observer, and the update of the estimated states mainly depended on the mode switching and the measured output. In order to verify the performance of the designed observer, the proposed method was applied on the Beijing Jingkai expressway, and the analysis results demonstrate that the proposed method was able to accurately estimate the traffic density. However, because the observer was designed for the central structure only, it is difficult to apply the observer to large scale expressway networks. From the view of practical application, the structure of distributed observers should be studied for large-scale traffic networks in future work.

## Figures and Tables

**Table 1:** Results of cell partition.

Link	Cell	Length	Link	Cell	Length
1	1	230 m	3	8	260 m
	2	230 m		9	260 m
2	3	246 m	4	10	270 m
	4	246 m		11	270 m
	5	246 m	5	12	230 m
	6	246 m		13	230 m
	7	246 m	6	14	260 m

**Table 2:** Mean Percentage Error (MPE) of synchronous state observer.

Cell Number	Cell 1	Cell 2	Cell 3	Cell 4	Cell 5	Cell 6	Cell 7
RMSE	0.0096	0.047	0.067	0.018	0.0096	0.0085	0.011
Cell Number	Cell 8	Cell 9	Cell 10	Cell 11	Cell 12	Cell 13	Cell 14
MPE	0.0087	0.034	0.0097	0.026	0.032	0.0079	0.025
Mean Value of MPE			0.022				

Note: RMSE: Root Mean Squared Error.

**Table 3:** MPE of state jump observer.

Cell Number	Cell 1	Cell 2	Cell 3	Cell 4	Cell 5	Cell 6	Cell 7
RMSE	0.0083	0.026	0.033	0.015	0.0091	0.0073	0.009
Cell Number	Cell 8	Cell 9	Cell 10	Cell 11	Cell 12	Cell 13	Cell 14
MPE	0.0081	0.028	0.0086	0.019	0.029	0.0068	0.023
Mean Value of MPE			0.016				

**Table 4:** RMSE of synchronous state observer.

Cell Number	Cell 1	Cell 2	Cell 3	Cell 4	Cell 5	Cell 6	Cell 7
RMSE	1.48	3.39	3.92	1.84	2.08	1.76	2.00
Cell Number	Cell 8	Cell 9	Cell 10	Cell 11	Cell 12	Cell 13	Cell 14
RMSE	2.36	2.85	1.73	2.27	2.99	1.65	2.30
Mean Value of RMSE		2.33					

**Table 5:** RMSE of state jump observer.

Cell Number	Cell 1	Cell 2	Cell 3	Cell 4	Cell 5	Cell 6	Cell 7
RMSE	1.34	2.29	2.42	1.28	1.42	1.66	1.73
Cell Number	Cell 8	Cell 9	Cell 10	Cell 11	Cell 12	Cell 13	Cell 14
RMSE	2.34	2.79	1.62	1.91	2.35	1.18	1.92
Mean Value of RMSE			1.88				
